# Prions Adhere to Soil Minerals and Remain Infectious

**DOI:** 10.1371/journal.ppat.0020032

**Published:** 2006-04-14

**Authors:** Christopher J Johnson, Kristen E Phillips, Peter T Schramm, Debbie McKenzie, Judd M Aiken, Joel A Pedersen

**Affiliations:** 1 Program in Cellular and Molecular Biology, University of Wisconsin Madison, Madison, Wisconsin, United States of America; 2 Department of Animal Health and Biomedical Sciences, School of Veterinary Medicine, University of Wisconsin Madison, Madison, Wisconsin, United States of America; 3 Molecular and Environmental Toxicology Center, University of Wisconsin Madison, Madison, Wisconsin, United States of America; 4 Department of Soil Science, University of Wisconsin Madison, Madison, Wisconsin, United States of America; University of Toronto, Canada

## Abstract

An unidentified environmental reservoir of infectivity contributes to the natural transmission of prion diseases (transmissible spongiform encephalopathies [TSEs]) in sheep, deer, and elk. Prion infectivity may enter soil environments via shedding from diseased animals and decomposition of infected carcasses. Burial of TSE-infected cattle, sheep, and deer as a means of disposal has resulted in unintentional introduction of prions into subsurface environments. We examined the potential for soil to serve as a TSE reservoir by studying the interaction of the disease-associated prion protein (PrP^Sc^) with common soil minerals. In this study, we demonstrated substantial PrP^Sc^ adsorption to two clay minerals, quartz, and four whole soil samples. We quantified the PrP^Sc^-binding capacities of each mineral. Furthermore, we observed that PrP^Sc^ desorbed from montmorillonite clay was cleaved at an N-terminal site and the interaction between PrP^Sc^ and Mte was strong, making desorption of the protein difficult. Despite cleavage and avid binding, PrP^Sc^ bound to Mte remained infectious. Results from our study suggest that PrP^Sc^ released into soil environments may be preserved in a bioavailable form, perpetuating prion disease epizootics and exposing other species to the infectious agent.

## Synopsis

Transmissible spongiform encephalopathies (TSEs) are a group of incurable diseases likely caused by a misfolded form of the prion protein (PrP^Sc^). TSEs include scrapie in sheep, bovine spongiform encephalopathy (“mad cow” disease) in cattle, chronic wasting disease (CWD) in deer and elk, and Creutzfeldt-Jakob disease in humans. Scrapie and CWD are unique among TSEs because they can be transmitted between animals, and the disease agents appear to persist in environments previously inhabited by infected animals. Soil has been hypothesized to act as a reservoir of infectivity, because PrP^Sc^ likely enters soil environments through urinary or alimentary shedding and decomposition of infected animals. In this manuscript, the authors test the potential for soil to serve as a reservoir for PrP^Sc^ and TSE infectivity. They demonstrate that PrP^Sc^ binds to a variety of soil minerals and to whole soils. They also quantitate the levels of protein binding to three common soil minerals and show that the interaction of PrP^Sc^ with montmorillonite, a common clay mineral, is remarkably strong. PrP^Sc^ bound to Mte remained infectious to laboratory animals, suggesting that soil can serve as a reservoir of TSE infectivity.

## Introduction

Transmissible spongiform encephalopathies (TSEs, prion diseases) are a group of fatal neurodegenerative diseases that affect a variety of mammalian species and include bovine spongiform encephalopathy (BSE, “mad cow” disease), chronic wasting disease (CWD) of deer and elk, sheep scrapie, and Creutzfeldt-Jakob disease in humans [1]. The agricultural, economic, and social impacts of prion diseases have been intensified by evidence suggesting transmissibility of BSE to humans [2]. The putative infectious agent in these diseases, designated PrP^Sc^, is a misfolded isoform of the normal cellular prion protein (PrP^C^). The amino acid sequences of PrP^Sc^ and PrP^C^ are identical [3]; normal and abnormal forms of the protein differ only in conformation. No differences in posttranslational covalent modification have been demonstrated [3]. Circular dichroism and infrared spectroscopy indicate that the disease-specific isoform has a higher β-sheet and lower α-helix content than PrP^C^ [4]. The normal isoform is soluble and primarily monomeric in solution, whereas PrP^Sc^ forms insoluble aggregates.

Sheep scrapie and cervid CWD are unique among TSEs, because epizootics can be sustained by horizontal (animal-to-animal) transmission [5,6]. Routes of natural transmission remain to be clarified, but available evidence indicates that an environmental reservoir of infectivity contributes to the maintenance of these diseases in affected populations [6–8]. The expanding range of CWD (several regions of North America and Korea) increasingly brings domestic livestock, companion animals, and wildlife species into contact with infected animals and carcasses, and shedded TSE agent, raising the possibility of cross-species transmission. This was demonstrated by the recent detection in Colorado, USA, of a free-ranging, CWD-infected moose, a species not previously known to be affected by the disease in the wild [9].

Although other modes of environmental transmission of scrapie and CWD have been proposed (e.g., flesh flies [10], hay mites [11]), several lines of evidence point to soil as a reservoir for TSE infectivity. TSE infectivity exhibits remarkable resistance to inactivation by most chemical agents, radiation, and heat [12] and has been shown to persist after burial in soil for at least 3 y [13]. Anecdotal observations of healthy sheep contracting scrapie after occupying fields previously containing diseased animals have been reported [7,8]. Although these older studies did not account for the genetic susceptibility of the sheep under study, they suggest that scrapie agent can persist in the environment for years. Recent controlled field experiments provide more compelling evidence of the environmental persistence of prions. Miller et al. [14] demonstrated that naïve mule deer could contract CWD when housed in paddocks previously inhabited by infected animals or containing decomposed infected carcasses.

TSE agents directly enter the environment when carcasses of infected animals decompose [13], through alimentary shedding of the agent from gut-associated lymphoid tissue [15,16], or from urinary excretion from infected, nephritic animals [17]. Furthermore, bovine, sheep, and deer TSE agents have been introduced to soil environments through the burial of diseased carcasses and other infected material [18]. Animals ingest soil both deliberately and incidentally [19]. Cattle, deer, sheep, and other animals can consume hundreds of grams of soil daily [20,21]. Taken together, these data support the notion that PrP^Sc^-contaminated soil may allow intraspecies TSE transmission and enhance the likelihood of spread to other species. As a first step toward understanding the role of soil as a reservoir of TSE infectivity, we investigated the binding of PrP^Sc^ to common soil minerals and whole soils and examined the infectivity of mineral-bound prions.

## Results

### Binding of PrP^Sc^ to Soil Minerals

We examined the sorption of purified PrP^Sc^ to three common soil minerals (Table S1): quartz, montmorillonite (Mte, an expandable layered silicate clay mineral), and kaolinite (Kte, a nonexpandable phyllosilicate mineral). Quartz of two particle sizes was employed in sorption experiments: fine sand (hydrodynamic diameter [*d*_h_] = 125–250 μm), representing quartz concentrated in the sand and silt fractions of soils, and microparticles (*d*_h_ = 1–5 μm), representing quartz present in the coarse clay fraction [22]. Purified PrP^Sc^ (~0.2 μg) was introduced into aqueous suspensions (pH 7.0) of each soil mineral and subjected to 2-h mixing. Unbound PrP^Sc^ was separated from bound protein by centrifugation through a 750-mM sucrose cushion. Bound and unbound fractions were analyzed by SDS-PAGE and immunoblotting.

**Figure 1:**
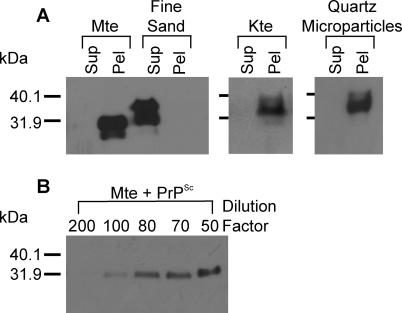
PrP^Sc^ Adsorption to Clay Minerals and Quartz Microparticles Substantially Exceeded That to Fine Quartz Sand (A) Detectable amounts of PrP^Sc^ adsorbed to Mte and Kte but not to fine quartz sand (*d*_h_ = 125–250 μm). PrP^Sc^ desorbed from Mte was of lower molecular mass than the starting material. Adsorption to quartz was observed when quartz microparticles (*d*_h_ = 1–5 μm) were employed and surface area was matched to Mte. (B) Immunoblotting sensitivity was determined by dilution of Mte-adsorbed PrP^Sc^ to the limit of detection. Protein was desorbed from Mte in 50 μl of SDS-PAGE sample buffer at 100 °C and serially diluted. Immunoblots used monoclonal antibody (mAb) 3F4. Pel, PrP^Sc^ associated with pelleted mineral particles; Sup, unbound PrP^Sc^ in supernatant.

The extent of PrP^Sc^ sorption differed among the mineral particles examined. All detectable PrP^Sc^ adsorbed to the expandable clay mineral Mte (Figure 1A). X-ray diffraction analysis provided no evidence that PrP^Sc^ entered Mte interlayer spaces (Mte *d*_001_ spacings were 1.22 nm and 1.47 nm before and after PrP^Sc^ adsorption, respectively); prion protein appeared to adsorb to only external clay surfaces. PrP^Sc^ did not associate with an equal mass of fine quartz sand at levels detectable by immunoblotting (Figure 1A). A large degree of PrP^Sc^ binding to the nonexpandable clay mineral Kte was observed when the surface area was matched to that of external Mte surfaces (Figure 1A). The limited association of PrP^Sc^ with fine quartz sand was at least in part attributable to the much smaller specific surface area of these particles as compared to kaolinite and external Mte surfaces (Table S1). When quartz surface area was matched to that of external Mte surfaces, all detectable PrP^Sc^ adsorbed to quartz (Figure 1A).

### Adsorption Capacities of Soil Minerals for PrP^Sc^

The amount of PrP^Sc^ adsorbed to Mte was semiquantitatively assessed by serial dilution of samples to the limit of immunoblotting detection. The dilution at which no detectable immunoreactivity remained provided a basis for comparison with samples lacking immunoreactivity before dilution. PrP^Sc^ desorbed from Mte still exhibited immunoreactivity after 100-fold dilution, indicating that the amount of prion protein adsorbed to Mte exceeded that in samples without immunoreactivity (e.g., unbound PrP^Sc^ in experiments with Mte) by at least two orders of magnitude (Figure 1B). Furthermore, this result suggests that fine quartz sand was saturated by at least 100-fold less PrP^Sc^ (≤ 0.002 μg) than used for sorption experiments (Figure 1A).

To assess the PrP^Sc^-binding capacity of the other soil minerals, increasing quantities of PrP^Sc^ were added to each mineral. Protein desorbed from mineral particles was serially diluted and subjected to SDS-PAGE and immunoblotting to semiquantitate the amount of sorbed protein. The binding capacity of a mineral was attained when subsequent PrP^Sc^ additions did not further increase the dilution factor required to reach the limit of immunoblotting detection (Table 1). Of the minerals examined, Mte exhibited the highest PrP^Sc^ adsorption capacity (~100 μg_protein_ mg_Mte_^−1^). The adsorption capacity of the quartz microparticles was nearly 10-fold less (~15.6 μg_protein_ mg_microparticle_^−1^), and that of Kte was nearly 100-fold less than Mte (~2 μg_protein_ mg_Kte_^−1^). When expressed on a surface-area basis (Table 1), the adsorption capacities of Mte and quartz microparticles were indistinguishable by our measurement method; that of Kte was 25 times less. These data demonstrate that mineral surface properties contribute to differences in the amount of PrP^Sc^ bound.

**Table 1:**
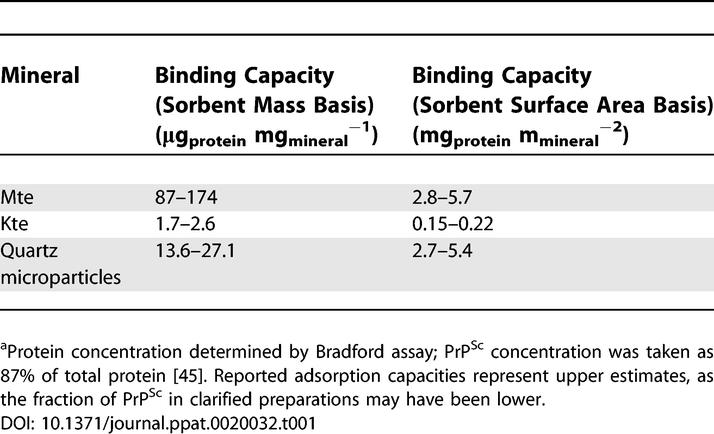
PrP^Sc^ Adsorption Capacities for the Minerals Examined^a^

### PrP^Sc^ Desorbed from Mte Surfaces Is Cleaved

Unexpectedly, PrP^Sc^ desorbed from Mte surfaces exhibited a lower molecular mass (~27–31 kDa) than the starting material (~33–35 kDa) (Figure 1A). Neither contaminant proteases nor metal oxide coatings on Mte particles appeared responsible for PrP^Sc^ cleavage, as treatments to counteract each did not prevent cleavage (unpublished data). Prior to sorption experiments, Mte was boiled in a solution of 10 mM NaCl for 10 min to denature contaminant proteases, or binding experiments were performed in the presence of a cocktail of protease inhibitors to inactivate them. Neither treatment prevented PrP^Sc^ cleavage. Amorphous metal oxide coatings on clay mineral particles can alter their surface reactivities and could potentially be responsible for PrP^Sc^ cleavage. The size-fractionated Mte used in this study has been reported to not contain such impurities at levels detectable by X-ray diffraction analysis [23], and precautionary pretreatment of the clay with a buffered neutral citrate-bicarbonate-dithionate solution to remove metal oxide coatings [24] failed to prevent cleavage.

**Figure 2:**
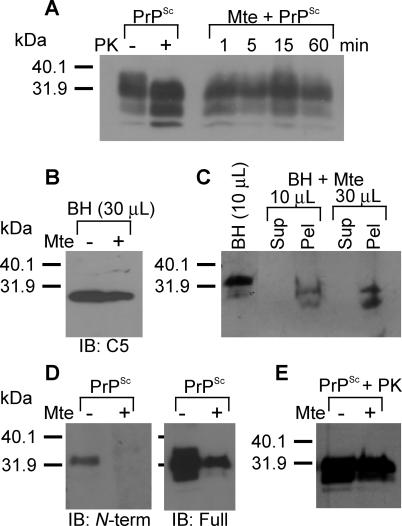
PrP^Sc^ Desorbed from Mte Is Cleaved (A) PrP^Sc^ cleavage occurs after short contact times with Mte surfaces. (B) The molecular mass protein C2 of the 20*S* proteasome subunit from BH was unaltered following desorption from Mte. (C) Cleavage of PrP^Sc^ present in infected BH was apparent after desorption from Mte. (D) PrP^Sc^ desorbed from Mte lost immunoreactivity against an antibody recognizing the N-terminal portion of the mature protein. (E) PrP^Sc^ pretreated with PK bound to Mte and did not exhibit further reduction in molecular mass when desorbed. Immunoblots (A, B, and E) used mAb 3F4. Immunoblots (C and D) employed anti-C2 and R20 polyclonal antibodies, respectively. Pel, PrP^Sc^ associated with pelleted mineral particles; Sup, unbound PrP^Sc^ in supernatant.

Prion protein desorbed from Kte and quartz did not exhibit a change in molecular mass (Figure 1A), suggesting that surface properties specific to Mte were responsible for the cleavage. Previous studies on protein interaction with Mte have not noted reductions in molecular mass upon desorption [25,26]. We incubated PrP^Sc^ with Mte for short time periods (1–15 min) to qualitatively investigate initial adsorption and cleavage kinetics. Adsorption of PrP^Sc^ to Mte was apparent within 1 min, and reduction in protein molecular mass was discernable (Figure 2A). Prion protein cleavage consistently occurred early within the first 15 min of contact with Mte and appeared maximal by 60 min. Cleavage of PrP^Sc^ caused by sorption to or desorption from Mte seemed to be a phenomenon specific to this protein. We examined sorption and desorption of scrapie-infected hamster brain homogenate (BH) to Mte. Desorption of brain proteins from Mte produced no changes in the overall molecular mass distribution as visualized by Coomassie blue staining (unpublished data). Subunit C2 of the 20S proteasome (~29 kDa), an unrelated protein similar in size to PrP likewise did not appear cleaved upon desorption from Mte (Figure 2B). In contrast, PrP^Sc^ in BH was cleaved (Figure 2C).

Cleavage of PrP^Sc^ involved loss of the N-terminal portion of the protein, which is not necessary for infectivity [3]. Prion protein desorbed from Mte lost immunoreactivity with an antibody directed against amino acids 23–37 on the protein N terminus, indicating that all or part of the epitope of this antibody was missing from the desorbed protein (Figure 2D). In contrast, probing identical samples with a polyclonal antibody against full-length PrP demonstrated that PrP^Sc^ was desorbed from the Mte. Although the precise cleavage site was not determined, these data suggest that the N terminus of PrP^Sc^ was removed; the fate of the cleaved amino acid residues is not known, as they may have remained bound to the clay or may have been extracted but not detected. When the N-terminal ~70 amino acids were removed from PrP^Sc^ by pretreatment with proteinase K (PK) prior to adsorption to Mte, we observed sorption to the Mte, but no further reduction in molecular mass upon desorption, evidence that other regions of the protein remain intact when associated with Mte (Figure 2E). These results also indicate that the N terminus of PrP^Sc^ is not necessary for adsorption to Mte.

### Strength of PrP^Sc^ Binding to Mte

**Figure 3:**
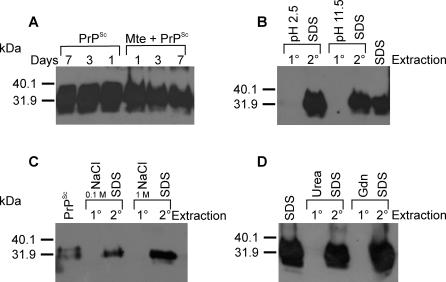
PrP^Sc^ Adsorbed to Mte Avidly and Remained Stable (A) PrP^Sc^ was stable when adsorbed to Mte for at least 7 d. (B) Extremes in pH (100 mM phosphate at pH 2.5 or 11.5), (C) sodium chloride (100 mM or 1 M), and (D) chaotropic agents (8 M urea or 8 M guanidine [Gdn]) did not desorb detectable amounts of PrP^Sc^ from Mte. Primary extractions (1°) were followed by secondary extractions (2°) extractions with a 10% SDS solution at 100°C. Immunoblots (A–D) employed mAb 3F4. Pel, PrP^Sc^ associated with pelleted mineral particles; Sup, unbound PrP^Sc^ in supernatant.

PrP^Sc^ attachment to Mte was avid, and sorbed PrP^Sc^ was stable. Washing Mte-PrP^Sc^ with the background solution used in sorption experiments did not induce detachment of detectable amounts of PrP^Sc^ from Mte (unpublished data). Contact of PrP^Sc^ with Mte for up to 1 wk did not result in additional degradation, indicating that the protein was not rendered more susceptible to cleavage by further structural rearrangements on the clay surface (Figure 3A). The strength of PrP^Sc^ attachment to Mte was surprising, even in light of reports of protein sorption-desorption hysteresis on mineral surfaces [26]. Conditions previously employed to desorb other proteins from soil minerals were largely ineffective in detaching PrP^Sc^ from Mte surfaces [26,27]. In our experiments, described above, a solution containing 10% SDS at 100 °C was used to remove the PrP^Sc^ from mineral surfaces. Changes in pH often alter interactions between clay surfaces and sorbed proteins [27,28]. Incubation of Mte-bound PrP^Sc^ in 100 mM phosphate buffer at pH 2.5 or 11.5, proton activities substantially higher and lower than the reported isoelectric points for PrP^Sc^ [29], failed to release the protein (Figure 3B). Likewise, increases in ionic strength (0.1 M or 1 M NaCl) failed to remove detectable PrP^Sc^ from Mte (Figure 3C). Strong chaotropic agents can be effective in desorbing proteins from soil minerals by disrupting hydrogen bonds [26]; however, neither 8 M urea nor 8 M guanidine released detectable amounts of PrP^Sc^ from Mte (Figure 3D). Our data indicate the interaction between PrP^Sc^ and Mte is strong and of high affinity.

### PrP^Sc^ Bound to Mte Remains Infectious

Sorption of proteins to soil particles often results in structural rearrangements that cause loss or diminution of function [25,27,30]. If binding to Mte surfaces results in (partial) unfolding of PrP^Sc^, a reduction or loss of infectivity would be expected, as denaturation renders the protein non-infectious [31]. We therefore tested whether PrP^Sc^ adsorbed to Mte remained infectious by intracerebrally inoculating hamsters with Mte-PrP^Sc^ complexes (Table 2). The time to onset of clinical symptoms after inoculation provides a measure of infectivity [32]. Hamsters inoculated with Mte-PrP^Sc^ exhibited clinical symptoms of scrapie 93 dpi. To control for any unbound prion protein that may have cosedimented with Mte particles, mineral-free PrP^Sc^ suspensions were processed in the same manner as in sorption experiments. The sedimented fraction of these control samples (mock pellets) showed substantially less infectivity than Mte-PrP^Sc^ pellets with a mean incubation period of 178 d, 105 d longer than Mte-PrP^Sc^ pellets. Hamsters inoculated with supernatants from these control samples (mock supernatants) showed clinical symptoms 103 dpi. Animals intracerebrally inoculated with Mte alone and uninoculated animals did not exhibit TSE symptoms during the course of the experiment (200 d).

**Table 2:**
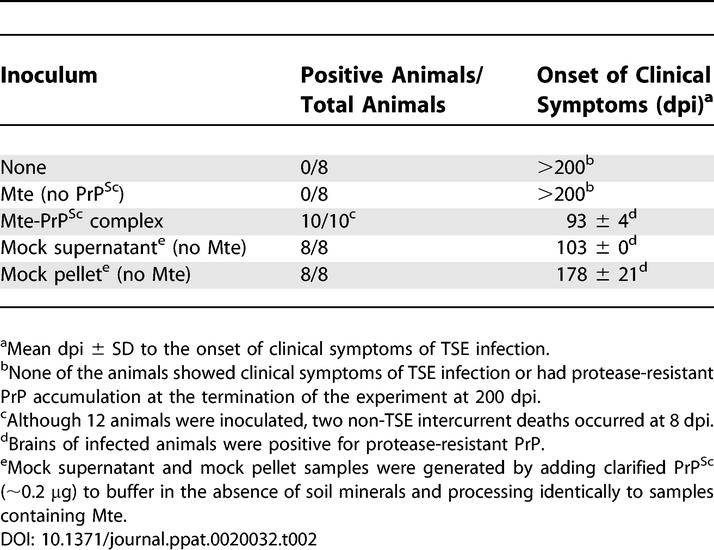
Prions Adsorbed to Montmorillonite Clay Retain Infectivity

### Whole Soils Bind PrP^Sc^

**Figure 4:**
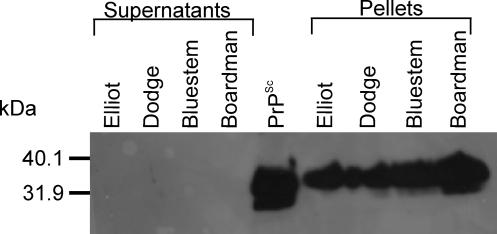
Whole Soils Bind PrP^Sc^ Elliot, Dodge, Bluestem, and Boardman soils bound PrP^Sc^ (pelleted soils). No immunoreactivity (i.e., no unbound PrP^Sc^) was detected in the supernatants. Immunoblot employed mAb 3F4.

To examine the extent of prion protein binding by whole soils, we conducted PrP^Sc^ sorption experiments with four soils differing in texture and mineralogy (Table S2). When equal masses of soil (0.5 μg) were used, all soils bound PrP^Sc^ to a similar extent (Figure 4); no detectable PrP^Sc^ remained in the supernatant at the level of protein used in this experiment. Prion protein desorbed from the soils did not appear cleaved. Several nonmutually exclusive factors may have contributed to this finding, including (1) relatively small amounts of Mte in some samples, (2) occlusion of Mte cleavage sites by metal oxide and/or natural organic matter coatings, and (3) competition among the various sorption domains (both inorganic and organic) for PrP^Sc^, limiting interaction with Mte. The amount of immunoreactive PrP^Sc^ recovered from each soil differed slightly; for example, the immunoreactive protein desorbed from the Elliot soil was less than that from the Boardman soil. This may have been due to stronger interaction of PrP^Sc^ with the Elliot soil than with the Boardman soil, leading to incomplete extraction, consistent with the larger fraction of clay-sized particles in the Elliot soil (Table S2).

## Discussion

Environmental transmission of prion diseases has been noted for decades [7,8,14]. In this study, we provide evidence indicating that soil and soil minerals serve as a reservoir of TSE infectivity. While extrapolation of in vitro studies to the environment must be made with caution, our findings suggest that PrP^Sc^ released from diseased animals may be sequestered near the soil surface, maintaining the TSE agent in an environmental medium with which livestock and wildlife come in contact. Our experiments demonstrate that Mte-bound PrP^Sc^ remains infectious and suggest that soil may harbor more TSE agent than previously assumed on the basis of water extraction of prions from garden soil [13].

Our results demonstrate that all soil mineral surfaces examined bound PrP^Sc^ and that Mte and quartz have larger specific binding capacities for PrP^Sc^ than does Kte (Figure 1). Although not relevant to TSE transmission, nonglycosylated, recombinant PrP^C^ has been shown to bind to Mte [33]. Interestingly, the N terminus of PrP^Sc^ desorbed from Mte was truncated (Figures 1A and 2). While Mte is known to catalyze several reactions, including the deamination of free glutamine and aspartic acid [34] and the polymerization of RNA into oligomers [35], protease activity has not been noted previously. The interaction between Mte and PrP^Sc^ is remarkably avid, as the only extractant used in this study that effected desorption was a solution containing 10% SDS at 100 °C (Figure 3B–3D). Prion protein appears unlikely to readily desorb from Mte in the environment. The propensity for PrP^Sc^ to tenaciously bind to Mte could be exploited in landfills to isolate prion-infected materials and prevent migration of the infectious agent.

The observation that prions remained infectious when bound to Mte is intriguing in light of the results of the desorption experiments; PrP^Sc^ adsorbed to Mte was extremely difficult to remove. Current mechanistic models for conversion of PrP^C^ to the pathological form require direct PrP^C^–PrP^Sc^ interaction [36]. The brain is unlikely to possess microenvironments capable of extracting significant amounts of PrP^Sc^ from clay surfaces. The 10-d increase in incubation period for Mte-adsorbed PrP^Sc^ relative to clay-free controls (mock supernatant) was statistically significant (*p* < 0.05) and would correspond to approximately a 1-log increase in infectivity [32]. This result suggests that PrP^Sc^-Mte complexes are inherently more infectious than the unbound protein and/or adsorption to Mte reduces clearance from the brain. We consider it likely that PrP^Sc^ adsorbed to Mte surfaces was available to convert PrP^C^ in the brain to the pathological isoform. Our findings are reminiscent of reports in which metal wires exposed to scrapie agent harbored significant infectious agent despite attempts to remove attached PrP^Sc^ [37,38].

The infectivity of soil- and soil mineral-sorbed PrP^Sc^ following oral exposure warrants investigation. The binding of PrP^Sc^ to soil particles could reduce oral bioavailability such that soil serves as a sink rather than a reservoir for infectivity. Conversely, association with mineral particles may protect the agent from degradation in the gastrointestinal tract, possibly enhancing transmission [39]. For example, bovine rotaviruses and coronaviruses retain infectivity via the oral route when bound to clay minerals [40]. While desorption of the protein from soil particles is more likely to occur in the gut than in the brain, removal of PrP^Sc^ from mineral particles may not be necessary to initiate infection.

In conclusion, soil and soil minerals have the potential to bind PrP^Sc^ and maintain infectivity. These findings will serve as the basis for further study on the interaction of PrP^Sc^ with other soil components (humic substances, quartz, and other minerals), the stability of soil-bound PrP^Sc^ under typical environmental conditions (UV light, freeze-thaw cycles) and the effect of soil microorganisms and extracellular enzymes on protein integrity. Our current results suggest that sorption of PrP^Sc^ to clay minerals may limit its migration through the soil column. Maintenance of prion infectivity at the soil surface may contribute to the propagation of CWD and scrapie epizootics and enhance the likelihood of interspecies transmission of these diseases.

## Materials and Methods

### Preparation of soil minerals and soils.

Montmorillonite (SWy-2) and kaolinite (KGa-1b) clays, obtained from the Clay Minerals Society Source Clays Repository (West Lafayette, Indiana, United States), were size-fractionated by wet sedimentation to obtain particles with *d*_h_ = 0.5–2 μm and saturated with sodium. These reference clay samples were extensively characterized previously [23,41]. Fine quartz sand (*d*_h_ = 125–250 μm) and SiO_2_ microparticles (*d*_h_ = 1–5 μm; 99% purity) were obtained from Sigma (St. Louis, Missouri, United States). The fine quartz sand was soaked for 24 h in 12 N HCl to remove impurities. X-ray diffraction analysis and infrared photoacoustic spectroscopy indicated that the SiO_2_ microparticles were composed of quartz.

We examined PrP^Sc^ sorption to four soils (Table S2). The Elliot soil was a silty clay loam purchased from the International Humic Substances Society (St. Paul, Minnesota, United States). Organically amended Dodge soil (sandy clay loam) was obtained from a glaciated upland area in Madison, Wisconsin. The Bluestem soil was a sandy clay loam collected from a fluvial deposit in Cedar Rapids, Iowa. The Boardman soil was a silt loam taken from an eolian deposit in Boardman, Oregon. Characteristics of these soils are presented in Table S2.

### Source of PrP^Sc^.

Syrian hamsters (cared for according to all institutional animal care and handling protocols of the University of Wisconsin, Madison) were experimentally infected with the Hyper strain of hamster-adapted transmissible mink encephalopathy agent. PrP^Sc^ was purified to a P_4_ pellet from brains of infected hamsters by a modification of the procedure described by Bolton et al. [42,43]. The P_4_ pellet prepared from four brains was resuspended in 1 ml of 10 mM Tris (pH 7.4) with 130 mM NaCl. For experiments employing PK-treated PrP^Sc^, 20% brain homogenate was treated with 50 μg ml^−1^ of proteinase K for 30 min at 37 °C. After blocking PK activity with 5 mM phenylmethylsulfonyl fluoride, purification was performed as above.

### Batch sorption experiments.

Larger prion aggregates were removed from purified PrP^Sc^ by collecting supernatants from two sequential 5-min centrifugations at 800 *g* (clarification step). Clarified PrP^Sc^ (~0.2 μg) was added to 500 μg of Mte or fine quartz sand, 1,500 μg of Kte, or 3.2 mg of quartz microparticles in 10 mM NaCl buffered to pH 7.0 with 10 mM 3-*N*-morpholinopropanesulfonic acid (MOPS) (500 μl final volume). In some cases, Mte experiments were conducted in unbuffered 10 mM NaCl. Sorption experiments with Mte performed in buffered and unbuffered 10 mM NaCl yielded comparable results. Experiments with Mte, Kte, and quartz microparticles each employed equivalent (external) mineral surface areas. In sorption experiments with whole soil samples, ~2 μg of clarified PrP^Sc^ was added to 5-ml suspensions of each soil (5 mg) in 5 mM CaCl_2_. Samples were rotated at ambient temperature for 2 h or an indicated time period. Sorption appeared complete within 2 h, as longer incubation times did not result in changes in levels of bound protein.

Each PrP^Sc^-mineral suspension and a 500-μl aliquot of each PrP^Sc^-soil suspension was placed over a 750 mM sucrose cushion prepared in a solution of the same composition as the background solution in the sorption experiment, and centrifuged at 800 *g* for 7 min to sediment mineral or soil particles and adsorbed PrP^Sc^. A sucrose cushion was found necessary to prevent a fraction of unbound PrP^Sc^ from sedimenting during centrifugation. Clarified PrP^Sc^ did not sediment through the sucrose cushion (Figure S1).

Unbound PrP^Sc^ remaining in the supernatant was precipitated with four volumes of cold methanol and resuspended in SDS-PAGE sample buffer (100 mM Tris [pH 8.0], 10% SDS, 7.5 mM EDTA, 100 mM dithiothreitol, and 30% glycerol). PrP^Sc^ was extracted from pelleted mineral particles with SDS-PAGE sample buffer at 100 °C for 10 min. The same procedure was followed for PrP^Sc^-soil suspensions. To determine mineral adsorption capacities for prion protein, varying volumes of clarified PrP^Sc^ preparation were added to a 1:100 dilution of each mineral suspension. All adsorption experiments were repeated at least three times.

For BH sorption experiments, 10% BH was clarified by collecting supernatants from two sequential 5-min centrifugations at 800 *g*. Aliquots (10 or 30 μl) of clarified BH were rotated with Mte in 10 mM NaCl at ambient temperature for 2 h; complexes of Mte and BH constituents were then sedimented through a sucrose cushion and processed as described in the preceding paragraphs.

All samples prepared for SDS-PAGE were separated on 4%−20% precast gels (BioRad, Hercules, California, United States) under reducing conditions. Proteins were transferred to polyvinyl difluoride membranes and immunoblotted with mAb 3F4 (1:40,000 dilution), R20 N-terminal pAb (1:10,000 dilution), Rab 9 pool 2 full-length PrP pAb (1:10,000 dilution), or anti-20*S* proteosome subunit C2 pAb (1 μg ml^−1^; A.G. Scientific, San Diego, California, United States). Detection was achieved with an HRP-conjugated goat anti-mouse immunoglobulin G (IgG) (BioRad) for mAb 3F4 and an HRP-conjugated goat anti-rabbit IgG (BioRad) for all pAbs.

### X-ray diffraction analysis.

PrP^Sc^ preparation (10 μg) was added to 50 μg of Mte in 10 mM NaCl (final volume of 0.5 ml). Samples were rotated at ambient temperature for 2 h and centrifuged at 16,100 *g* for 7 min. After centrifugation, the bulk of the supernatant was removed, leaving a small amount of solution above the clay pellet. The clay was resuspended in the remaining supernatant, and the slurry was placed on silica wafer slides and stored in a desiccator for over 12 h. The basal *d*_001_ spacings of near homoionic Na^+^-SWy-2 before and after adsorption of PrP^Sc^ were determined by X-ray diffraction on a Scintag PAD V diffractometer (Cupertino, California, United States) using CuKα radiation and continuous scanning from 3° to 15° 2θ with a step size of 0.02° and a dwell time of 2 s.

### Extraction experiments.

PrP^Sc^ adsorbed to Mte was incubated for 30 min at room temperature in 8 M urea or 8 M guanidine HCl (50 μl per pellet), 0.1 or 1 M NaCl (25 μl per pellet), or 100 mM sodium phosphate (pH 2.5 or 11.5; 25 μl per pellet). Primary extractions with these solutions were followed by secondary extractions with SDS-PAGE sample buffer at 100 °C to assess the efficacy of the primary extraction. Urea and guanidine primary extracts were dialyzed against double distilled water for 2 h (nominal molecular weight cutoff, 12–14 kDa; Fisher Scientific, Pittsburgh, Pennsylvania, United States) prior to SDS-PAGE analysis.

### Infectivity bioassay.

PrP^Sc^-Mte pellets prepared as above were resuspended in pH 7.4 PBS (50 μl per pellet) and intracerebrally inoculated into male, weanling Syrian hamsters (Harlan, Indianapolis, Indiana, United States). Equivalent amounts of PrP^Sc^ starting material or Mte without PrP^Sc^ were inoculated into control animals. Hamsters were monitored every 3 d for the onset of clinical symptoms [32,44]. Brains from clinically positive hamsters and uninfected controls were analyzed for protease-resistant PrP by immunoblotting.

## Supporting Information

Figure S1 Sucrose Cushion Prevented Sedimentation of Unbound PrP^Sc^ under Conditions Necessary to Pellet Soil Minerals A substantial amount of unbound PrP^Sc^ pelleted when centrifuged under conditions required to remove Na^+^-Mte from suspension, but was prevented from sedimenting by a sucrose cushion. Sucrose cushions were therefore employed in batch sorption experiments to prevent sedimentation of unbound PrP^Sc^. Results from representative mock adsorption experiments are shown. PrP^Sc^ was rotated in a solution of 10 mM NaCl in the absence of soil minerals for 2 h and was either placed above a 750 mM sucrose cushion and centrifuged (two right lanes), or centrifuged without a sucrose cushion (two left lanes). Supernatants (Sup) and pellets (Pel) were analyzed by immunoblotting with mAb 3F4.
(17 KB PDF)

Table S1 Characteristics of Minerals Used in PrP^Sc^ Sorption Experiments (25 KB DOC)

Table S2 Characteristics of Soils Used in PrP^Sc^ Sorption Experiments (26 KB DOC)

### Accession Numbers

The GenBank (http://www.ncbi.nlm.nih.gov/) accession number for PrP^Sc^ is M14054.

## Abbreviations

BH: brain homogenate
BSE: bovine spongiform encephalopathy
CWD: chronic wasting disease
dpi: days postinoculation
Kte: kaolinite
Mte: montmorillonite
PK: proteinase K
PrP^C^: normal cellular isoform of the prion protein
PrP^Sc^: disease-associated prion protein
TSE: transmissible spongiform encephalopathy
