# Supplementary material for: Prions Adhere to Soil Minerals and Remain Infectious
Source: PLoS Pathog. 2006 Apr 14;2(4):e32. doi: 10.1371/journal.ppat.0020032 (PMC1435987; doi:10.1371/journal.ppat.0020032)
Supplement: Supplementary file 1 [file ppat.0020032.sg001.pdf]

Figure S1.

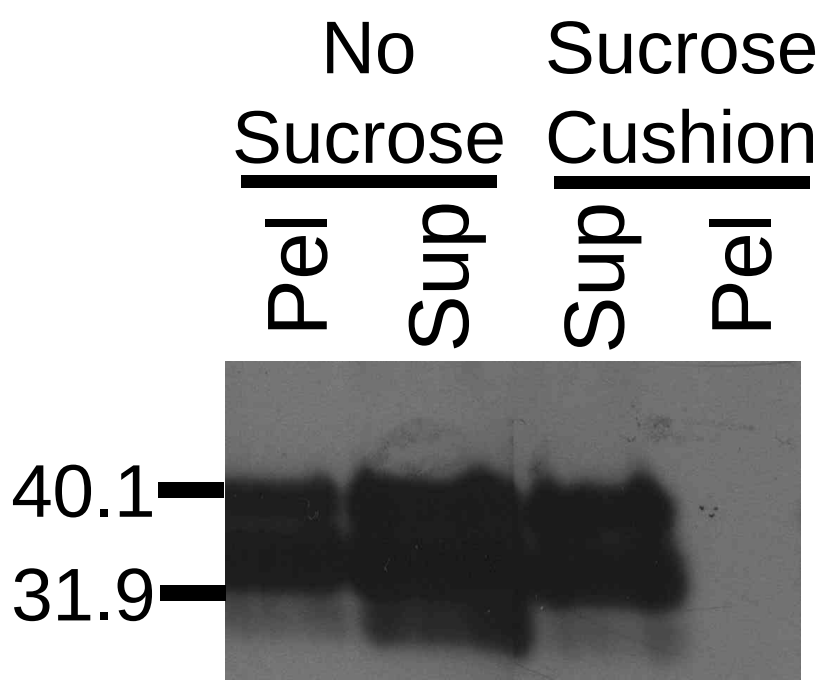

Figure S1. Sucrose cushion prevented sedimentation of unbound PrP^{Sc} under conditions necessary to pellet soil minerals. A substantial amount of unbound PrP^{Sc} pelleted when centrifuged under conditions required to remove Na^{+}-Mte from suspension, but was prevented from sedimenting by a sucrose cushion. Sucrose cushions were therefore employed in batch absorption experiments to prevent sedimentation of unbound PrP^{Sc}. Results from representative mock adsorption experiments are shown. PrP^{Sc} was rotated in a solution of 10 mM NaCl in the absence of soil minerals for 2 h and was either placed above a 750 mM sucrose cushion and centrifuged (two right lanes), or centrifuged without a sucrose cushion (two left lanes). Supernatants (Sup) and pellets (Pel) were analyzed by immunoblotting with mAb 3F4.
