# Supplementary material for: Prions Adhere to Soil Minerals and Remain Infectious
Source: PLoS Pathog. 2006 Apr 14;2(4):e32. doi: 10.1371/journal.ppat.0020032 (PMC1435987; doi:10.1371/journal.ppat.0020032)
Supplement: Supplementary file 2 [file ppat.0020032.st001.doc]

**Table S1** Characteristics of minerals used in PrP^Sc^ sorption experiments.

| **Mineral** | **Effective Hydrodynamic Diameter**  **(*μ*m)** | **Specific Surface Area**^†^  **(m^2^·g^-1^)** | **CEC**^‡^  **(cmol_(+)_·kg^-1^)** | **pH_pzc_^║^** |
| --- | --- | --- | --- | --- |
| Na^+^-Mte | 0.5-2.0 | 31.8^*^ | 76.1 ± 3.4 | 2.5 [1, 2] |
| Na^+^-Kte | 0.5-2.0 | 11.7 | 1.52 ± 0.07 | 4.7 [1, 2] |
| Fine Quartz Sand | 125-250 | 0.04 | 0.28 ± 0.01 | 2.9 [3, 4] |
| Quartz Microparticles | 1-5 | 5.0 | 0.64 ± 0.04 | 2.9 [3, 4] |

^†^ External surface areas were measured by N_2_ adsorption for clay minerals and quartz microparticles (Brunauer-Emmet-Teller (BET) method [5]); surface area of the fine quartz sand was determined by Kr adsorption (BET method).

^*^ Total (internal + external) specific surface area was measured by ethylene glycol monoethyl ether adsorption and found to be 749.5 ± 18.9 m^2^·g^-1^. X-ray diffraction data indicated the internal surface area was not accessible to PrP^Sc^.

^‡^ Cation exchange capacity (CEC) was determined by compulsive exchange with Ba^2+^ [6].

^§^ Surface charge density

^║^ pH of point of zero charge

# References

1.	Parks GA (1965) The isoelectric points of solid oxides, solid hydroxides and aqueous hydroxy complex system. Chem. Rev. 65: 117-198.

2.	Davis JA (1982) Adsorption of natural dissolved organic matter at the oxide/water interface. Geochim Cosmochim. Acta 46: 2381-2393.

3.	Sverjensky DA, Sahai N (1996) Theoretical prediction of single-site surface-protonation equilibrium constants for oxides and silicates in water. Geochim. Cosmochim. Acta 60: 3773-3797.

4.	Sahai N, Sverjensky DA (1997) Evaluation of internally consistent parameters for the triple-layer model by the systematic analysis of oxide surface titration data. Geochim. Cosmochim. Acta 61: 2801-2826.

5.	Carter DL, Mortland MM, Kemper WD (1986) Specific surface. In: *Methods of soil analysis, part 1. Physical and mineralogical methods*. 2nd ed. Arnold Klute, A. L. Page, editors. Madison, Wis.: American Society of Agronomy : Soil Science Society of America. p 413-423.

6.	Sumner ME, Miller WP (1996) Cation exchange capacity and exchange coefficients. In: *Methods of soil analysis: Part 3. chemical methods*. 1st ed. D. L. Sparks, editor. Madison, Wis.: American Society of Agronomy : Soil Science Society of America. p 1201-1229.
