# Supplementary material for: Prions Adhere to Soil Minerals and Remain Infectious
Source: PLoS Pathog. 2006 Apr 14;2(4):e32. doi: 10.1371/journal.ppat.0020032 (PMC1435987; doi:10.1371/journal.ppat.0020032)
Supplement: Supplementary file 3 [file ppat.0020032.st002.doc]

**Table S2** Characteristics soils used in PrP^Sc^ sorption experiments.

| **Parameter** | **Elliot Soil** | **Dodge Soil** | **Bluestem Soil** | **Boardman Soil** |
| --- | --- | --- | --- | --- |
| specific surface area^†^ (m^2^·g^-1^) | 10 | 3.6 | 10.3 | 13 |
| ƒ_oc_^‡^ | 0.029 | 0.34 | 0.0042 | 0.0047 |
| **Soil texture** | | | | |
| % sand | 7 | 55 | 50 | 37 |
| % silt | 62 | 25 | 23 | 55 |
| % clay | 31 | 30 | 27 | 8 |
| **Mineralogy (mass percent)**^*^ | | | | |
| quartz | 71 | 63 | 67 | 34 |
| K-feldspar | 3.6 | 6.6 | 6.9 | 6.3 |
| plagioclase | 8.6 | 15 | 15 | 35 |
| amphibole | 2.4 | 0.8 | 2.5 | 1.7 |
| calcite | 0.3 | 0.5 | 0.4 | 2.3 |
| dolomite | 1.8 | 4.1 | – | – |
| hematite | – | – | 1.2 | 0.5 |
| mixed-layer illite/smectite | 14^§^ | 16^║^ | 4.4^║^ | 5.9^║^ |
| illite + mica | 8.7 | 6.4 | 1.3 | 10 |
| kaolinite | 1.7 | 2.0 | 1.7 | 1.7 |
| chlorite | 0.4 | 0.1 | 0.2 | 2.7 |

^†^ Specific surface area measured by N_2_ adsorption (BET method [1])

^‡^ Mass fraction of organic carbon determined by the organic carbon dry combustion method using a Leco CNS-2000 (St. Joseph, MI).

^*^ Determined by x-ray diffraction analysis (K/T Geoservices Inc., Arglye, TX)

^§^ Ordered mixed-layer illite/smectite with 40% smectite layers. Mte is a smectite clay mineral.

^║^ Randomly ordered mixed-layer illite/smectite with 90% smectite layers

# References

1.	Carter DL, Mortland MM, Kemper WD (1986) Specific surface. In: *Methods of soil analysis, part 1. Physical and mineralogical methods*. 2nd ed. Arnold Klute, A. L. Page, editors. Madison, Wis.: American Society of Agronomy : Soil Science Society of America. p 413-423.
